# Annexins—a family of proteins with distinctive tastes for cell signaling and membrane dynamics

**DOI:** 10.1038/s41467-024-45954-0

**Published:** 2024-02-21

**Authors:** Volker Gerke, Felicity N. E. Gavins, Michael Geisow, Thomas Grewal, Jyoti K. Jaiswal, Jesper Nylandsted, Ursula Rescher

**Affiliations:** 1 Institute of Medical Biochemistry, Center for Molecular Biology of Inflammation (ZMBE), University of Münster, Von-Esmarch-Strasse 56, Münster, Germany; 2 Department of Life Sciences, Centre for Inflammation Research and Translational Medicine (CIRTM), Brunel University London, Uxbridge, UK; 3 The National Institute for Medical Research, Mill Hill, London, UK; 4 Delta Biotechnology Ltd, Nottingham, UK; 5 School of Pharmacy, Faculty of Medicine and Health, University of Sydney, Sydney, NSW Australia; 6 Center for Genetic Medicine Research, Children’s National Research Institute, Children’s National Research and Innovation Campus, Washington, DC, USA; 7 Department of Genomics and Precision Medicine, The George Washington University School of Medicine and Health Sciences, Washington, DC, USA; 8 Danish Cancer Institute, Strandboulevarden 49, Copenhagen, Denmark; 9 Department of Molecular Medicine, University of Southern Denmark, J.B. Winsløws Vej 21-25, Odense, Denmark; 10 Research Group Cellular Biochemistry, Institute of Molecular Virology, Center for Molecular Biology of Inflammation (ZMBE), University of Münster, Von-Esmarch-Strasse 56, Münster, Germany

**Keywords:** Cell biology, Calcium, Drug discovery, Membrane lipids

## Abstract

Annexins are cytosolic proteins with conserved three-dimensional structures that bind acidic phospholipids in cellular membranes at elevated Ca^2+^ levels. Through this they act as Ca^2+^-regulated membrane binding modules that organize membrane lipids, facilitating cellular membrane transport but also displaying extracellular activities. Recent discoveries highlight annexins as sensors and regulators of cellular and organismal stress, controlling inflammatory reactions in mammals, environmental stress in plants, and cellular responses to plasma membrane rupture. Here, we describe the role of annexins as Ca^2+^-regulated membrane binding modules that sense and respond to cellular stress and share our view on future research directions in the field.

## Abstract (web summary)

Annexins are calcium-regulated membrane binding proteins with an array of cellular activities. Here, Gerke et al. describe recent research highlighting the many functions of annexins and provide a view on directions for the future.

## Annexins—emergence of a multifaceted protein family

The first member of the annexin (Anx) protein family was discovered by biochemical approaches that, as we now know, built upon the unique membrane-binding properties of annexins. In search of Ca^2+^-regulated factors that would promote exocytosis of catecholamine-containing granules in chromaffin cells of the adrenal medulla, Carl Creutz and Harvey Pollard identified a soluble factor that bound to Ca^2+^, interacted with chromaffin granule membranes, mediated their aggregation, and thus, promoted the meeting of membrane surfaces in exocytotic membrane fusion. They aptly named this factor “synexin” derived from the Greek “synexis”^1^. Additional “synexin-like” proteins, later shown to be annexins, were then identified^2^ suggesting already at this point that this group of proteins participates in membrane bridging, docking, and/or fusion processes. Independently, lipotropin-1 or lipocortin-1, now known as annexin A1, was discovered as a phospholipase inhibitor protein that mediates the anti-inflammatory actions of glucocorticoids^3^.

**Fig. 1:**
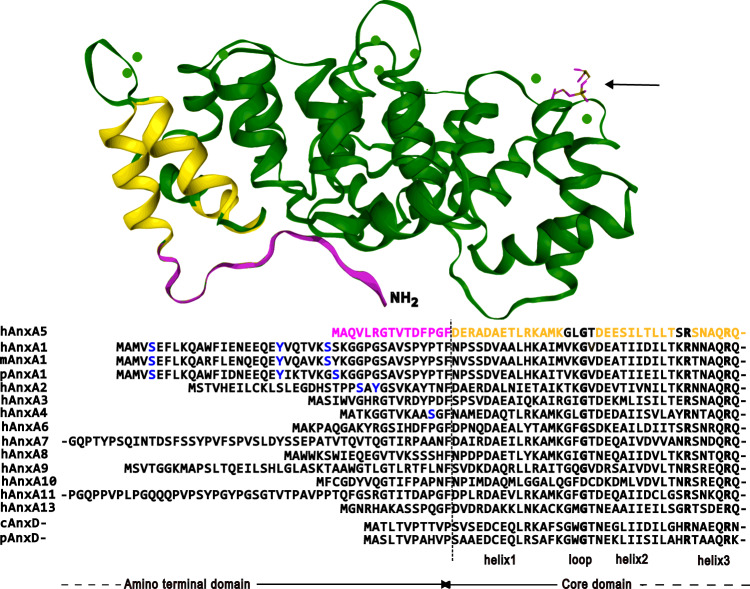
Annexin structural organization. Illustrated (top) is the 3D structure of annexin 5 (Protein Data Bank IA8A; Swairjo et al.^13^). It is used here as an example of the canonical annexin fold. Green circles (size exaggerated) indicate the location of bound Ca^2+^ ions. The annexin ligand indicated by an arrow is a glycerophosphoserine molecule—here representing the membrane lipid phosphatidylserine. The sequence data and some amino acid annotations below the 3D structure have been taken from the UniProt database. This currently contains over 30,000 annexin sequences, but selected regions of human (h), mouse (m), and pig (p) AnxA1 and two plant kingdom annexins from (1) cotton (cAnxD) and (2) a bell pepper (pAnxD) are included for comparison as evolutionary distant proteins. The sequences have initially been aligned by the CLUSTAL program. Selected homologous and/or known invariant residues are shown in bold font. Key serine (S) and tyrosine (Y) phosphorylation sites are shown in blue. From inspection of the Protein Structure Data Base (PDB), residues in the unstructured amino-terminal domains have been highlighted in magenta and those within the secondary structures at the commencement of the annexin core domain (helices 1–2 and part of helix 3) are highlighted in yellow. The corresponding regions of the annexin 5 structure shown at the top of the figure have been analogously colored. There are no experimental 3D structures for AnxA7, 9, 10, or 11 and so helices of the other annexins have not been highlighted in the illustration, but can be found in the PDB or can be inferred from strong sequence homologies. To accommodate the alignments in an accessible figure, the long amino termini of hAnx7 and 11 have been truncated. Similarly, the sequences within the annexin core domain have been arbitrarily interrupted within the third helix. Were the complete core domain sequences to be aligned in this manner, this would demonstrate analogous alignments of the 4 or (uniquely 8 for AnxA6) sequence repeats of ~70 residues which constitute the cores of all annexins.

Contemporaneously, around a dozen different groups reported the isolation of analogous Ca^2+^-dependent membrane-binding proteins from diverse vertebrate tissues and leukocytes. Limited peptide analyses revealed highly homologous amino acid sequence repeats across these proteins^4^ and this was confirmed by cDNA sequencing, initially of annexin A1^5^. An annexin-specific common tertiary structure, the so-called Anx “core”, then emerged in 3D structures, starting with X-ray crystallography of human annexin A5^6^. This core contains four (in annexin A6, eight) annexin repeats, segments around 70 amino acids in length that each fold into five α-helices connected by short loops or turns (Fig. 1). The entire core domain adopts the form of a compact disc, with a distinctly convex surface that interacts with cell membranes and a concave surface that is available for interactions within the cytosol. In contrast to the uniform core domain, the annexin N-terminal domains are unique. They emerge at the concave side of the core and interact with cytosolic factors (see below). The recognition of these proteins as members of a family possessing common biochemical properties, the Ca^2+^-dependent binding to acidic phospholipids, and homologous 3D molecular structures led to the adoption of the present generic name annexins (Anx): proteins that can associate with membranes. Annexins were initially identified in vertebrates, and through work mainly in mammals, it is now well-established that they are expressed to different extents in different tissues and cells (Table 1).

**Table 1:** Annexin expression patterns and link to diseases

Annexin	Expression		Disease
	Cells	Tissues	
A1	Most cells; highly prominent in differentiated cells, e.g., macrophages, neutrophils; low in T lymphocytes	Most tissues; high in nervous and endocrine system; extracellular occurrence; low in adaptive immune system	**Acute/chronic inflammation** E.g., rheumatoid arthritis, multiple sclerosis, asthma, lung fibrosis, bacterial infection, bowel disease, Systemic Lupus Erythematosus (SLE), viral infection (influenza) **Cardiovascular disease** Stroke, atherosclerosis, type 1 + 2 diabetes, restenosis, myocardial infarction **Tissue/membrane repair** Muscle injury, intestinal and skin wound repair **Central nervous system** Epilepsy, Alzheimer’s disease, cerebral ischemia **Cancer** E.g., breast and pancreatic cancer, fibrosarcoma (NSCLC), myeloma, nasopharyngeal carcinoma
A2	Most cells; abundant in endothelial cells, monocytes, macrophages	Most tissues; high in lung, pancreas, colon, ileum and adrenal tissues; low in spleen, testis, kidney, liver	**Vascular homeostasis and angiogenesis** E.g., acute carotid artery injury, diabetic retinopathy, stroke, atherosclerosis **Pulmonary vasculature** E.g., lung injury, pulmonary fibrosis and inflammation **Vascular homeostasis** E.g., cerebral venous thrombosis, antiphospholipid syndrome **Lipid disorders** Hypercholesterolemia, diabetes, obesity, metabolic syndrome **Cancer** E.g., pancreatic ductal adenocarcinoma (PDAC), breast, ovarian, NSCLC, lung, acute lymphoblastic leukemia (ALL), multiple myeloma, fibrosarcoma **Neurological disorders** Alzheimer’s disease **Muscle repair** Dysferlinopathy, Limb girdle muscular dystrophy (LGMD) 2B
A3	Many cell types	Many tissues; preferentially spleen, lung and reproductive organs	**Cancer** Ovarian, breast, pancreatic, hepatocellular, lung, bone **Pain** Bone cancer induced pain
A4	Most cells; prominent in many secretory epithelial cells. Three AnxA4 mRNAs (a,b,c): AnxA4a broadly distributed; AnxA4c only in solitary chemosensory cells	Most tissues; prominent in secretory epithelia of lung, intestine, stomach, kidney. AnxA4b mRNA only in digestive track.	**Cancer** Gallbladder, breast, platinum resistance in endometrial carcinoma, ovarian cancer and plaxitaxel resistance in lung cancer **Other** Cardiac function, lung development, skin wound repair
A5	Most cells; most abundant annexin in many cells, except neurons	Most tissues; high and most abundant annexin in most tissues; substantial extracellular amounts	**Anticoagulation** E.g., pregnancy loss, thrombosis, blood vessel injury **Wound repair and inflammation** Skin wound repair, lung fibrosis and inflammation, muscular dystrophy **Cardiovascular disease** Myocardial infarction (MI), non-alcoholic steatohepatitits (NASH) **Cancer** E.g., hepatocellular carcinoma, renal cell carcinoma, melanoma **Diagnostic use** Detection of apoptosis in cancer models, liver and vascular apoptosis, efferocytosis, immune tolerance; detection of cancer cell death after chemotherapy **Therapeutic use** Cardiovascular disease treatment
A6	Most cells; abundant in endothelial cells, endocrine cells, hepatocytes, macrophages; low/undetectable in epithelial cells of small intestine, colon and parathyroid gland	Most tissues; high in skeletal muscle, liver, heart, spleen, lymph nodes	**Cardiac function** Cardiomyopathy, heart failure **Bone and cartilage** Osteoarthritis, pain in osteoarthritis **Lipid and liver disorders** Liver regeneration and dysfunction, insulin resistance **Cancer** E.g., breast cancer, epidermal growth factor receptor (EGFR)-related cancers; PDAC, gastric cancer **Membrane repair** Muscle dystrophy
A7	Two isoforms (47 and 51 kD) in most cells	47 kD isoform in most tissues, except skeletal muscle; 51 kD isoform in heart, brain and myotubes	**Pancreatic function** Beta-cell hypertrophy, insulin secretion **Cardiovascular disease** Cardiomyocyte contraction, heart arrythmia, cardiac remodeling, atherosclerosis **Brain** Astrocyte proliferation, neuronal apoptosis
A8		Low abundance in lung, liver, kidney, skin, placenta, cornea	Possible role in preimplantation period during pregnancy
A9		Low abundance mainly in fetal and adult liver and spleen	No AnxA9-KO mouse model. Autoantibodies in *Pemphigus vulgaris*
A10	Epithelial cells	Mainly in epithelia of gastrointestinal tract	No AnxA10-KO mouse model
A11	Ubiquitous	Ubiquitous	**Cancer** E.g., hepatocellular carcinoma **Immune disorders** Mutations in SLE and granulomatous disease **Brain** Amyotrophic lateral sclerosis (ALS), frontotemporal dementia

The table summarizes expression patterns of mammalian annexins based mainly on data obtained from animal models. Diseases possibly linked to overexpression or lack of the respective annexin are also listed. These data are mostly based on transgenic and knock-out mouse models with verification/application in humans. For reference, see refs. ^150^ and ^151^ and references therein.

The first identification of annexins in higher plant cells broadened the family’s taxonomy with plant genomes encoding between 8 and 25 non-redundant annexin genes^7–9^. Genome sequencing programs currently show annexin genes to be present in almost all eukaryotes. Not only multicellular but also free-living unicellular eukaryotes, e.g., protists, express multiple annexin paralogs. The annexin nomenclature was therefore extended to classify vertebrate (AnxA), invertebrate (AnxB), fungus (AnxC), plant (AnxD), and protist (AnxE) genes plus an identifying alphanumeric postscript (e.g., AnxA2 for vertebrate annexin family member 2). Even the possible discovery of prokaryotic annexins is covered: they would be classified as AnxF ‘in abeyance’. Annexin genes from mammals and plants or other eukaryotes differ substantially in sequence and expression control, e.g., the typical plant amino acid sequence homology with animal annexins is less than 50%^9^. Yet, despite separation from the animal lineage over evolutionary deep time and the very different cellular communities in which they are expressed, plant annexins retain analogous 3D structures, similar biochemical properties, and many of the intracellular activities described for animal annexins^8,9^. Only subtle variations can be observed, e.g., the presence of a sulfur cluster in cotton Anx that could contribute to the plant’s response to oxidative stress^10^. Annexin evolution thus elegantly exemplifies Darwin’s dictum of “descent with modification”.

However, even in the case of mammals, where annexin expression has been widely studied, several unanswered questions remain. Despite differential cell and tissue expression (Table 1), control of annexin expression and post-transcriptional processing remains an important area of future work. This is especially so due to the growing role of annexins in human pathologies. At the level of the genome, there is a need to understand both genetic and epigenetic regulation. To give just one example, important post-transcriptional control by non-coding nuclear RNAs has been reported. This was found especially significant in the case of AnxA1 and A2 where several microRNAs and long non-coding RNAs have been shown to suppress expression of these annexins^11^. On the other hand, annexins themselves appear to interact with and regulate the synthesis and translocation of RNAs and hence appear to participate in regulatory feedback loops. Further work is also needed on the regulation of alternative annexin pre-mRNA splicing. Isoforms arising from splicing within the annexin core appear to affect membrane binding, and amino-terminal splicing events (as occurs in mammalian AnxA2, A4, and A6) may be assumed to result in changes in annexin partner binding.

### The annexin core

The Anx core is largely made up of Ca^2+^/membrane-binding structural motifs. The Ca^2+^-binding sites are represented by two antiparallel alpha-helices connected by short loop-forming polypeptides and are known as type II Ca^2+^ sites to distinguish them from the classical type I (EF hand) fold^12^. These are exemplified in the AnxA5 structure shown in Fig. 1. In these type II sites, Ca^2+^ is liganded by carboxyl and carbonyl oxygens located in the loops protruding at the convex side of the Anx surface and also coordinates to phosphoryl moieties of acidic phospholipids in membrane-bound annexins^13^. This arrangement accounts for the lipid-binding specificity of annexins. Through the bridging Ca^2+^, they bind to negatively charged phospholipids, e.g., phosphatidylserine (PS), phosphatidic acid, and phosphoinositides, in particular phosphatidylinositol 4,5-bisphosphate^14^ (the location of a phosphatidylserine mimetic is depicted in Fig. 1). Minor amino acid sequence changes within Anx paralog type II motifs lead to changes in the affinity (or absence) of Ca^2+^ and/or membrane binding. Interestingly, the (peripheral) phospholipid-binding properties of annexins can also be regulated by the composition of the interacting target membranes. Specifically, cholesterol as a lipid affecting membrane fluidity has been shown to render the Anx-lipid interaction cooperative^15^. Another ligand binding to the cores of several, but not all annexins in the presence of Ca^2+^ is F-actin. AnxA2 was initially identified as an F-actin interacting protein^16^, a property later shown to be displayed by other annexins as well^17^. Although the precise nature of the F-actin binding site in the Anx core domain is not known, it has been reported to include a sequence residing at the C-terminus^18^. In interactions mediated via the core domain, several annexins have also been shown to bind certain RNAs and regulate their transport and translation^19^. The experimentally determined structures and high sequence homologies of annexin cores from many animal and even plant kingdom annexins suggest that the core annexin fold has been highly conserved throughout evolution: the surface area of the molecule which interacts with lipid bilayers is large and the nature of membrane lipids similar across eukaryotic organisms.

### The annexin amino termini

**Fig. 2:**
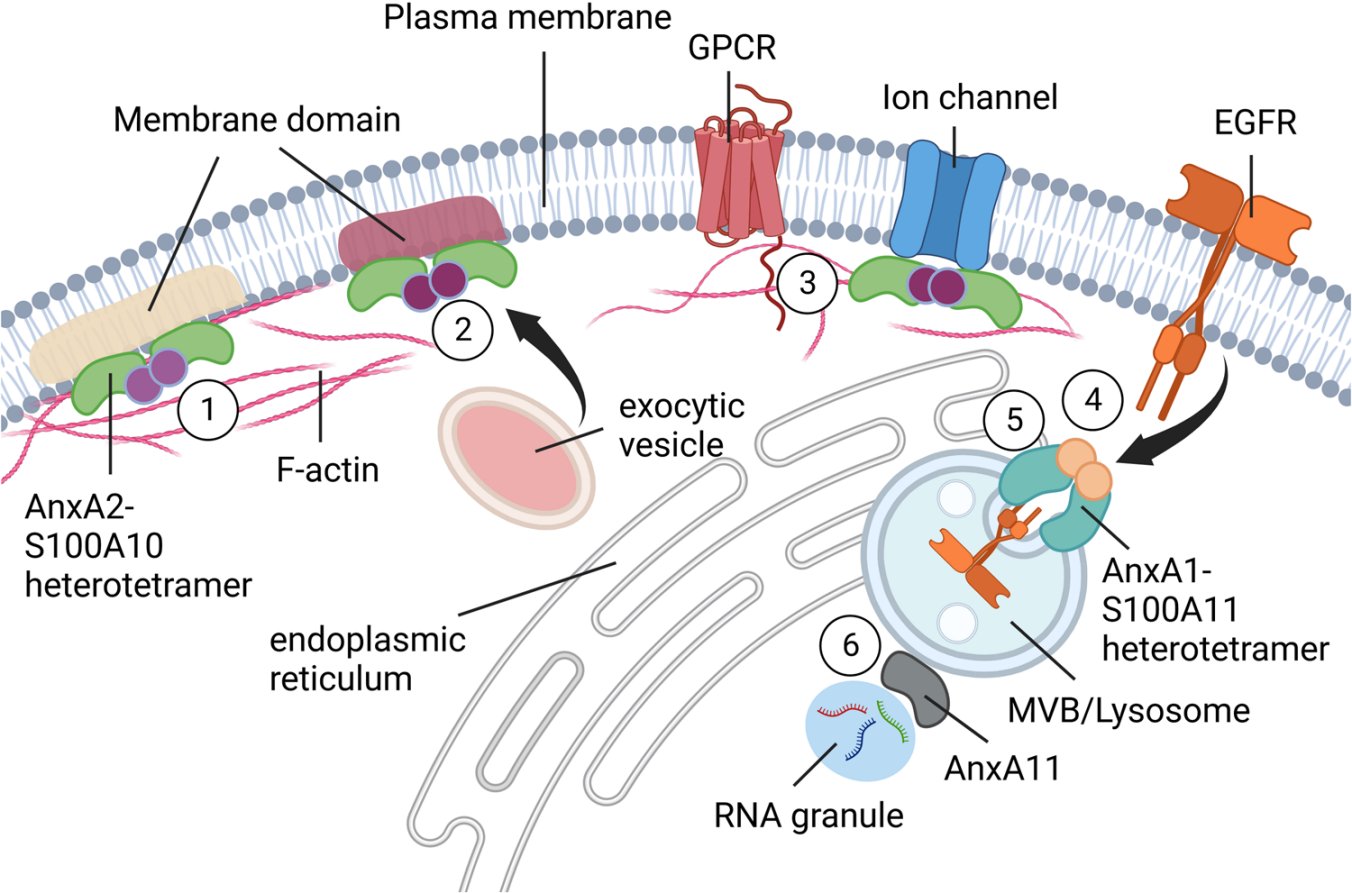
Assorted annexin functions at membrane interfaces. Examples are shown for AnxA1, AnxA2, and AnxA11. AnxA2 (green) in a heterotetrameric complex with S100A10 (purple) can coordinate plasma membrane domains that are enriched in negatively charged phospholipids such as phosphatidylserine (PS) and phosphatidylinositol 4,5-bisphosphate (PI(4,5)P_2_) and also cholesterol and are linked to cortical F-actin (1). The AnxA2-S100A10 heterotetramer also supports the fusion of exocytic vesicles by linking PI(4,5)P_2_-rich domains to tethering factors (2) and it interacts with ion channels (blue) and GPCRs (rose), positioning and anchoring them at the plasma membrane (3). AnxA1 (turquois) is a substrate of the activated growth factor receptor EGFR (orange), and the heterotetrameric complex consisting of AnxA1 and S100A11 (ocher) is involved in the sorting of EGFR onto luminal vesicles of multivesicular endosomes (4, 5) and the establishment of ER–endosome membrane contact sites. AnxA11 (gray) tethers membraneless RNA granules to mobile lysosomes (6). See text for references. Generated with BioRender.com.

The amino-terminal regions of Anx paralogs generally lack any homology and are considered to confer most of their specific properties and biological functions. However, the amino-terminal sequences of individual mammalian annexin homologs are highly similar, indicating related functions (see AnxA1 sequences in Fig. 1). These could involve a role of the amino-terminal segments as cleaved peptides (AnxA1) or as intrinsically misfolded domains undergoing phase separation (AnxA11, see below). A number of post-translational modifications occur within the amino-terminal segments and two of these have been highlighted in blue (tyrosine and serine phosphorylation sites) in Fig. 1. The amino termini also carry several recognition sequences for binding partner interactions—SH2 domain recognition through tyrosine phosphorylation and SH3 domain recognition—the longer annexin amino termini (not shown) are rich in proline residues. It is also noteworthy that known alternative splicing is especially prevalent in some annexin amino termini indicating control of or alternative function. Importantly, the N-terminal sequences of AnxA1 and AnxA2 form amphipathic alpha-helices that constitute binding sites for EF hand-type Ca^2+^-binding proteins of the S100 family. S100A10 is a canonical interaction partner of AnxA2. It forms a tight dimer and upon binding to the N-terminal domain of AnxA2, triggers the establishment of a heterotetrameric AnxA2-S100A10 complex^20,21^. Interestingly, the Ca^2+^ binding loops in S100A10 have suffered mutations rendering the protein permanently active with respect to binding AnxA2. A similar situation prevails in the AnxA1-S100A11 complex. However, Ca^2+^ binding to S100A11 is required to establish a conformation capable of interacting with AnxA1^22^. Anx complexes with other EF hand proteins, most notably between AnxA7 and sorcin^23^, have also been described but are structurally less well-defined. As the Anx-type membrane-binding sites are accessible in the Anx-S100 heterocomplexes, these can function in bridging two membrane surfaces thereby supporting tethering. However, for AnxA2-S100A10, another membrane-bound configuration has also been identified by atomic force microscopy of the complex bound to solid-supported bilayers^24^. Here, both AnxA2 subunits interact with acidic phospholipids in the membrane and the S100A10 dimer faces the aqueous environment, i.e., the cytosol in a cellular context, where it is accessible for additional protein interactions. Several such interactions have been identified, including membrane receptors and ion channels such as the serotonin receptor, Nav1.8 and TRPV5 and 6 ion channels^25–28^ and their binding motifs might resemble the amphiphatic alpha-helices found in AnxA1 and AnxA2. Hence, a role for the AnxA2-S100A10 complex as membrane-binding module that stabilizes membrane proteins at the plasma membrane and also links tethering factors to the exocytotic vesicle fusion machinery, Munc13-4 in the case of endothelial cell Weibel–Palade body exocytosis^29^, has emerged (Fig. 2). Interestingly, cortical AnxA1 was recently also shown to direct interaction partners to certain sites at the plasma membrane. In mammary epithelial cells, AnxA1, most likely in complex with S100A11, interacts with the cell polarity regulator Leucine–Glycine–Arginine repeat protein (LGN) to instruct a polarized localization of LGN (and other cell polarity proteins) at the lateral cell cortex. This in turn is required for proper orientation of a planar mitotic spindle and thus epithelial morphogenesis and lumen formation^30^. Whether such annexin-based complexes show an altered membrane-binding behavior, such as a preferred binding to lipid domains involved in exocytosis and cortical spindle anchorage is not known and remains to be determined.

## From structure to a multitude of membrane-related functions

Since the dawn of annexin research, roles in coordinating membrane-related events were inferred from the common denominator of annexin functions, namely their ability to bind to membranes at elevated Ca^2+^ levels, which rise in response to physiological stimuli as well as in cell stress scenarios. Adequately, the different annexins display graded Ca^2+^ sensitivities, which could explain why many annexins respond to supraphysiological elevated Ca^2+^ levels, e.g., those caused by cell membrane injury, to a different extent. Here, we will highlight central membrane-related functions of certain annexins rather than giving a comprehensive summary and we would like to refer the reader to excellent reviews on specific functional aspects related to the annexin family.

### Annexins—modulators of membrane organization and transport

The emerging picture is one of proteins that fulfill functions in both constitutive and facilitated endo- and exocytotic routes. This most likely occurs by organizing membrane domains for tethering and/or subsequent fusion with respective transport vesicles, as shown in particular for AnxA2 (Fig. 2). High-resolution analyses involving cryogenic and conventional electron microscopy (EM) showed that AnxA2 forms junctions between the membrane surfaces of model liposomes and isolated chromaffin granules^31^ and probably constitutes the fine strands observed between chromaffin granules and the plasma membrane of stimulated adrenal chromaffin cells^32^. Extending these earlier structural and biochemical approaches, a role for annexins in exocytosis, in particular Ca^2+^-regulated exocytosis, has been well documented, initially in chromaffin cells and later in other cell systems. Using permeabilized cells, live cell imaging and electrophysiological recordings of exocytotic events, AnxA2 was shown to promote tethering and fusion of various secretory granules (chromaffin granules, Weibel–Palade bodies in endothelial cells, lamellar bodies in alveolar epithelial cells) with the plasma membrane^33–37^. Additional evidence for a structural role of AnxA2 and other annexins, most notably AnxA1 and AnxA6, in providing and organizing membrane contacts and thereby aiding membrane transport in exocytosis and endocytosis has accumulated over the years and has been reviewed elsewhere^38,39^. Interestingly, it was shown that the Ca^2+^-dependent role of AnxA2 in exocytosis is further modulated by post-translational modifications. Phosphorylation/dephosphorylation of AnxA2 in the protein amino-terminal region (see Fig. 1) has been reported at different serine residues in response to nicotinic stimulation in chromaffin cells and adrenergic stimulation in endothelial cells and these events regulate the activity of the protein in supporting exocytosis of the respective granules^40,41^.

In addition to AnxA2 and AnxA7, other annexins have been linked to membrane transport steps along the biosynthetic/exocytotic route. Here, we highlight only AnxA13 as its expression pattern and function is uniquely cell-type specific. AnxA13, in particular the splice variant AnxA13b, is specifically expressed in intestinal epithelial cells and subject to amino-terminal myristoylation. This modified isoform associates with sphingolipid- and cholesterol-rich subdomains of the trans-Golgi network and is involved in the budding of such domains and their exocytotic transport to the apical domain of the polarized cells^42^, in line with the common theme of annexins functioning in organizing membrane domains for subsequent fusion and/or tethering them to the respective secretory vesicles.

Given their membrane-binding and sculpting properties, it is not surprising that annexins, in particular AnxA1, A2, and A6, have also been identified as regulators of endocytic transport. AnxA1 was initially described as a substrate of activated EGF receptors (EGFRs)^43^. It is phosphorylated by the internalized receptors at the stage of multivesicular bodies/late endosomes (MVB/LE)^44^ and is involved in the formation of EGF-induced intraluminal vesicles that contain the internalized EGFR (Fig. 2)^45^. At the LE membrane interface, AnxA1 also participates in cholesterol transport from the ER to MVBs/LEs. This becomes particularly relevant when low-density lipoprotein (LDL)-cholesterol levels are low in the endosomes. As a heterotetrameric complex with S100A11, AnxA1 aids the formation of ER–endosome membrane contact sites probably by providing physical tethers^46^. AnxA2 most likely also functions in endocytic processes however, here as a monomer and not in association with its ligand S100A10. Its role in early endosome (EE) fusion and multivesicular endosome biogenesis^47^ was mechanistically linked to the nucleation of actin patches on endosomes. This additionally requires the actin nucleation factor Spire1 to most likely support membrane remodeling associated with endosome maturation (e.g., the formation of membrane tubules)^48^.

AnxA6 also functions in the endocytic pathway, as well as in cell signaling and various other aspects of cellular homeostasis^49–51^. It serves a scaffolding function for assembling signaling proteins that contribute to endocytic receptor trafficking. AnxA6 was shown to act as a scaffold protein for protein kinase C (PKC) alpha and p120 GTPase activating protein (GAP), negative regulators of the EGFR/Ras/mitogen-activated protein kinase (MAPK) pathway, promoting the targeting of EGFR to lysosomes for degradation^52^. A scaffolding role for AnxA6 coupled to signaling events in the recycling compartment probably also exists, as timely coordination to recycle the Na^+^-coupled neutral amino acid transporter SNAT4 to the sinusoidal plasma membrane during metabolic stress triggered by partial hepatectomy is compromised in AnxA6-depleted hepatocytes^53^. In the late endosomal compartment, AnxA6 also functions in the control of cellular cholesterol homeostasis, which is coupled to the ability of cells to establish membrane contacts between LE/lysosomes and the ER^54^. AnxA6 overexpression induces LE cholesterol accumulation, leading to secondary cholesterol depletion in other cellular compartments such as ER, Golgi, and plasma membrane. This compromised cholesterol trafficking impairs caveolae formation and also results in mislocalization and dysfunction of several soluble N-ethylmaleimide-sensitive factor attachment protein receptors (SNAREs) responsible for fibronectin secretion and integrin recycling, respectively^55,56^. Mechanistically, the AnxA6-dependent cholesterol accumulation in late endosomes is linked to the AnxA6-mediated recruitment of the Rab7-GTPase activating protein TBC1D15 to late endosomes, which in turn promotes Rab7 inactivation^54^. It is interesting to note that AnxA2 has also been shown to control the activity of two small GTPases, the Rho family member Cdc42 and RhoA^57,58^. These examples extend the annexin function as lipid domain organizers to participation in complex molecular machines that regulate vesicle trafficking.

Recent findings indicate that some regulatory roles of annexins may be related to their ability to control the formation of membrane contact sites (MCS), possibly through FFAT (two phenylalanines in an acidic tract) motifs that have been identified in other late endosomal/lysosomal proteins as interaction sites for ER-resident VAMP (Vesicle Associated Membrane Protein) associated proteins (VAPs). FFAT motifs are found in AnxA1, A6, and A11 and a variation of the original FFAT (Phospho-FFAT motif) has been described for AnxA5 and A8. Given the ability of several annexins (AnxA1, A6) to modulate cholesterol transport across MCS between LE/lysosomes and the ER^46,54^, the recognition of FFAT-like motifs within these annexins by ER-associated VAPs or motile sperm domain-containing proteins (MOSPDs) could affect membrane contact site formation.

A key question concerning endosome-associated annexins is whether there is a gradient of annexins localized to different endosome subtypes and whether such distinct localization pattern might only emerge in certain (pathophysiological) conditions, probably associated with elevated intracellular Ca^2+^ levels.

### Annexins and plasma membrane wound repair

Mechanical or chemical stress commonly causes injury to plasma membranes, especially in mechanically active tissues, e.g., muscle, gut, epidermis, and vasculature, and in cells exposed to chemical and pathogenic agents. To repair these injuries and survive, cells autonomously repair the injured plasma membrane, and this process crucially depends on annexins^59–61^.

The link between annexins and plasma membrane repair was initially proposed two decades ago and has now developed into a growing topic of basic and translational research. AnxA1 and AnxA2 interact with dysferlin, a protein mutated in Limb Girdle Muscular Dystrophy, they localize to the plasma membrane injury site and regulate muscle cell membrane and tissue repair^62–66^. AnxA5 and AnxA6 are also involved in muscle and myofiber membrane repair^67,68^. AnxA6’s role in muscle cell membrane repair was first recognized in zebrafish and genetic mapping of a mouse model of severe muscular dystrophy. A splice site mutation in this gene was later found to impair myofiber repair and worsen the disease^69,70^. AnxA1, AnxA2, AnxA5, and AnxA6 accumulate at sites of focal laser injury together with dysferlin, and their altered accumulation compromises myofiber repair^65,71,72^. However, the analysis of annexins in muscle disease is complicated due to the multiple roles of these proteins. While annexins such as AnxA2 and AnxA6 are required for myofiber membrane repair, AnxA1 is dispensable for this but is involved in regenerating damaged myofibers^73,74^.

Annexins also facilitate the repair of cell membranes injured by pore-forming toxins (PFTs) such as streptolysin O (SLO)^75^. Interestingly, differences in Ca^2+^ sensitivity of distinct annexins cause their differential recruitment to the injured membrane. Repair of membrane lesions formed by PFTs involves injury-triggered cell membrane shedding and calcium-sensitive accumulation of the Endosomal Sorting Complex Required for Transport III (ESCRT III) complex^76,77^. While the ESCRT III complex mediates the actual scission and shedding of the damaged cell membrane segments, its injury-triggered accumulation is mediated by AnxA7. Ca^2+^ influx due to cell membrane injury triggers AnxA7 to bind another calcium-sensitive protein, apoptosis-linked gene-2 (ALG-2), which recruits the ESCRT III anchoring protein ALG-2-interacting protein X (ALIX) to the damaged membrane^78^. This cooperativity facilitates focal formation of the repair machinery in response to the wounding-induced high intracellular Ca^2+^ levels and is in tune with the mediocre affinities of the annexin-type Ca^2+^-binding sites.

The interactions of certain annexins with their obligate partner S100 proteins also facilitate cell membrane repair. The AnxA2-S100A10 heterotetramer interacts with the membrane repair protein AHNAK and this complex may orchestrate membrane repair^79^. A related AnxA2-S100A11 protein complex enables cell membrane repair by promoting a build-up of F-actin at the injury site, where it helps close the wound and excise the damaged portion of the injured membrane^80^. In endothelial cells, S100A11 enables accumulation of AnxA1 and A2 at the injury site and supports repair by Ca^2+^-dependent interaction with extended synaptotagmin 1 (E-Syt1) which helps tether the injured plasma membrane with the ER^81^.

**Fig. 3:**
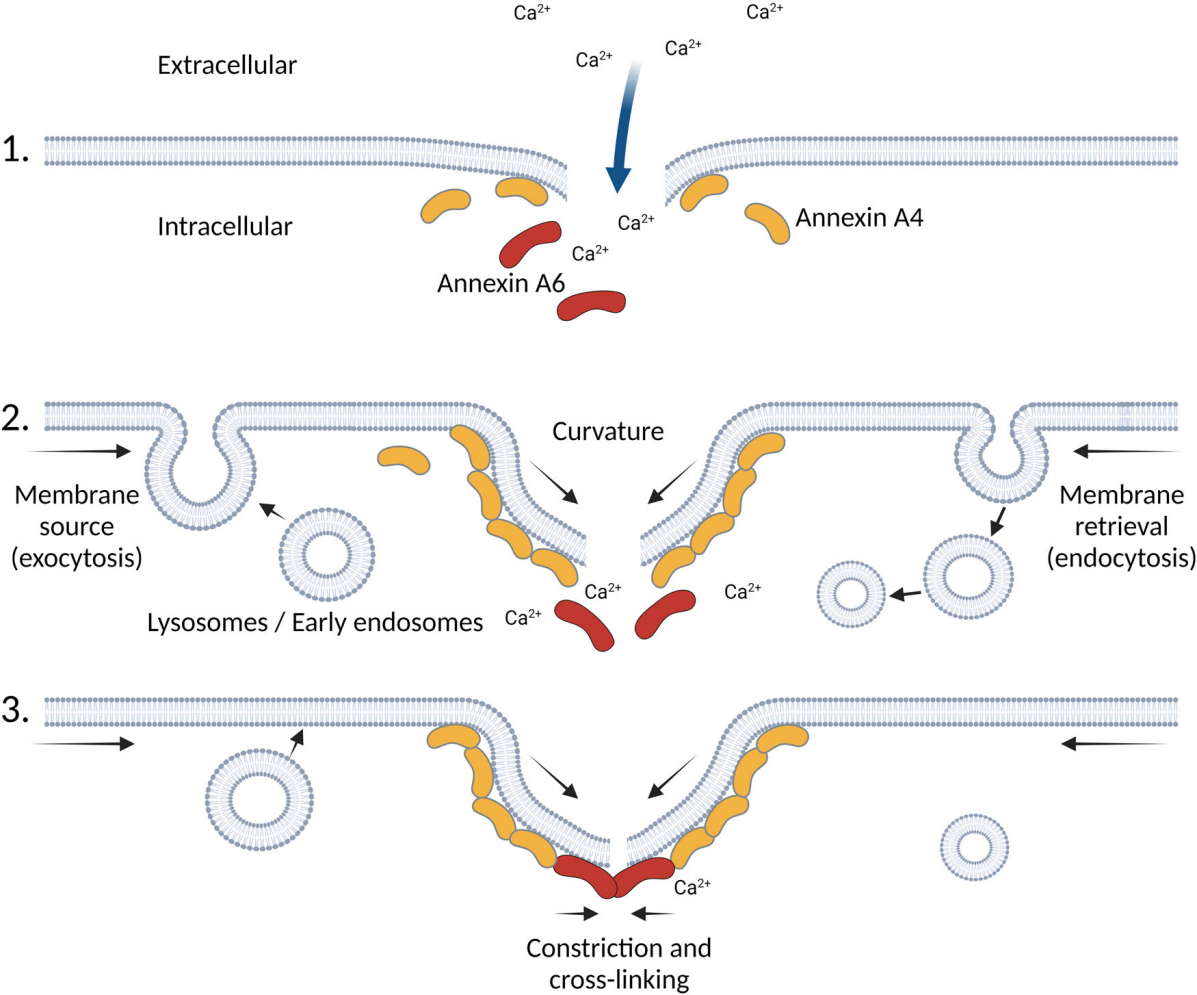
Annexins in plasma membrane repair. Ca^2+^ influx following plasma membrane injury triggers the recruitment of several annexins to the wound site. Working in concert, annexins such as AnxA4 then generate forces to curve the free edges of the injured membrane, while AnxA6 produces constriction forces along free membrane edges to eventually support the resealing (1). This is facilitated by the Ca^2+^-triggered exocytosis of early endosomes and lysosomes that likely provides additional membrane (2)^147–149^. Only AnxA4 and AnxA6 are shown here for simplicity. Other annexins recruited to the wound site and participating in repair are AnxA1 and AnxA2 which most likely support fusion, AnxA5 which provides a protein lattice inhibiting wound expansion, and AnxA7 which establishes a link to the ESCRT machinery (3, see the text for references). Generated with BioRender.com.

Similar to muscle and endothelial cells, the high motility of metastatic cancer cells also constantly exposes them to plasma membrane lesions. Accordingly, metastatic cancer cells routinely overexpress annexins, and silencing their expression impairs membrane repair and increases the death of invasive breast cancer cells^82^. Similarly, membrane intercalating drugs that compromise membrane binding of annexin, strongly sensitize cancer cells to membrane injury^83^. Recent biophysical studies of cancer cells and artificial, supported bilayers have provided additional mechanisms by which annexins mediate membrane repair. Importantly, it was shown that AnxA5 forms 2D protein arrays around the injury site, likely preventing further wound expansion^84^. This process is accompanied by an AnxA5-driven induction of lipid phase transition^85^. AnxA4 and A6, on the other hand, support membrane repair by directly binding the injured region of the cell membrane to help close the wound^86,87^. Working in concert, AnxA4 and AnxA5 generate forces to curve the free edges of the injured membrane by way of Ca^2+^-dependent homo-trimerization, while AnxA6 produces constriction forces along free membrane edges (Fig. 3)^86^. In line with this, induction of membrane curvature appears to be a common characteristic among the annexin family members based on experiments employing supported membrane models, with AnxA4 and A5 sensing highly curved membranes^87,88^. These findings suggest that membrane shaping and curvature sensing are interrelated through a feedback loop, where initial curvature generation by annexins triggers the recruitment of more annexins. Hence, through membrane shaping, aggregation, fusion, fission, and interaction with lipids and other calcium-sensitive proteins, annexins coordinate multiple processes to ensure membrane repair.

Although the above progress in the field of annexin research has rapidly expanded our current knowledge of annexin involvement in membrane repair we still have limited understanding of the interplay between the dual role of annexins as membrane tethers and membrane remodelers. Key questions remain to be answered. How do annexins differentiate different membrane repair processes, such as membrane tear versus toxin-induced holes? Is the supraphysiological Ca^2+^ level the only signal to direct annexins to the torn membrane? Do the lipid-binding profiles of annexins also regulate their response? Do they act in a concerted manner in supramolecular assemblies? In addition to formation of annexin lattices, can wound site-recruited annexins also form a plug or phase condensate to restrict diffusion through the injury site until the membrane-mediated resealing can occur?

## The great conundrum—extracellular annexin functions

Annexins are cytosolic proteins with no classical signal sequence guiding them to the secretory pathway. Nonetheless, some members of the family have been identified in the extracellular environment and extracellular functions have been assigned to several annexins. The arguably best-researched annexin in this regard is AnxA1, which dampens inflammatory reactions and leukocyte extravasation. Formyl Peptide Receptors (FPRs) were identified as the family of cell surface receptors through which extracellular AnxA1 is mediating its regulatory mode of action of transmitting anti-inflammatory and inflammation-resolving signals^89,90^. FPRs (of which there are three in humans: FPR1-3), are G-protein coupled receptors on leukocytes and other cells. They were initially described as sensors of bacterial formylated peptides, such as formyl–Methionine–Leucine–Phenylalanine (fMLF, formerly fMLP), that guide immune cells to sites of bacterial infections^91^. Meanwhile, these receptors are known to respond to a large cohort of chemically unrelated bacterial, viral, and endogenous ligands of the host^92,93^ and might function via biased signaling, i.e., tailored signaling depending on the ligand and cellular environment^94^. The FPR-binding domain in the AnxA1 molecule which had been mapped to the very N-terminal part of the molecule, is unique within the annexin family and the FPR interaction is therefore specific to AnxA1 and not shared with other annexins^89^.

Other annexins have also been reported to serve extracellular functions although in these cases specific receptors have remained mainly elusive. Rather, extracellular AnxA2 has been shown to act as an endothelial cell surface receptor for plasminogen. In the heterotetrameric complex with its ligand S100A10, AnxA2 drives the tissue plasminogen activator (tPA)-dependent activation of plasmin and thereby the degradation of fibrin. Cell surface AnxA2-S100A10 is therefore required to maintain vascular homeostasis and clear fibrin depositions, and reduced expression of AnxA2 has been shown to be associated with impaired cell surface fibrinolysis and venous thromboembolism^95–97^. Interestingly, the S100A10 subunit of the AnxA2-S100A10 complex was also reported to represent the tPA binding moiety involved in plasmin generation^98^. AnxA1 has also been implicated in thrombosis and thrombotic complications, including ischemic stroke (IS) and venous thromboembolism. Reduced AnxA1 levels were found in both, IS patients and mouse models for IS and administration of AnxA1 offered protection against IS damage (by its ability to reduce cerebral thrombosis, a prerequisite for stroke) and against subsequent thrombotic events post-stroke, an effect involving inside-out integrin signaling events in platelets^99^.

Another line of (extracellular) annexin biology identified the proteins as inhibitors of blood coagulation, however here as effectors of the coagulation cascade and not as plasminogen activators. Hence, the name vascular (or also platelet) anticoagulant protein was also used for some annexins in the early days of annexin research. Biochemical approaches isolated proteins with such anticoagulant activity that were later shown to bind to anionic phospholipids^100,101^. These lipids are present on the surface of activated platelets and endothelial cells, and annexin binding can render the phospholipids inaccessible to coagulation factors, thereby preventing their accumulation and activation.

Despite the extracellular functions reported for different annexins, their mode of secretion is still enigmatic, and the possible secretion or release of different annexins has not been compared in well-defined studies. One notable exception is an early work analyzing the extracellular occurrence of two family members, AnxA1 and AnxA4, in human seminal plasma. Whereas both proteins were shown to localize inside ductal epithelial cells of the prostate gland, only AnxA1 was present in the seminal fluid, indicative of a selective secretion process^102^. While the mechanism(s) underlying annexin release are not known, several externalization routes can be envisaged including neutrophil degranulation and exosomal secretion. ‘Penetration’ of the membrane through a transmembrane intermediate^103,104^ and the involvement of ABC transporters at the plasma membrane have also been discussed. Annexins can be released from injured cells through transient plasma membrane wounds (see above), and excessive AnxA2 released by this means can trigger muscle degeneration which is reversed by AnxA2 knockdown^105^. Interestingly, AnxA1 and AnxA6 have been found in extracellular vesicle fractions as palmitoylated proteins^106^. Although the underlying post-translational modification mechanisms are still unknown, these palmitoylated annexins may contribute to membrane contact site tethering within cells and retain those characteristics upon possible release in exosomes. Another post-translational modification, phosphorylation, has also been linked to the atypical annexin release from cells. In case of AnxA1, phosphorylation at serine-27 appears essential for secretion of the protein^107^ and tyrosine-23 phosphorylation of AnxA2 is a prerequisite for its release^108^. Thus, although evidence for a specific release exists for some annexins, the crucial question of how the proteins cross a membrane (be it an organelle membrane or the plasma membrane) and how post-translational modification affects this process is still unknown. Other important questions include: Are the release pathways unique to different annexins? Is externalization dependent on certain stimuli, a cell type, a cell stress condition? While addressing these questions is a fascinating topic in itself, at least from a cell biologist’s point of view, it might prove very relevant to clinical practice, be it in diagnosis or therapy, by using annexins as biological therapeutics or suppressing their release.

## Close encounters of the pathogenic kind—annexins and infections

Pathogens reliably detect their host cells through pathogenic surface compounds that bind to cognate receptors on the host cell surface, and the initial attachment either hinders (in the case of extracellular pathogens) or elicits (in the case of intracellular pathogens) entry into the host cell. Entry mechanisms often depend on the modulation of the cellular repertoire of uptake mechanisms to gain access to the host cell cytosol or to hide within the cell in membrane-bound vesicles, protected from the immune response. Thus, host-pathogen interactions require and cause the remodeling of cellular membranes and are often connected with changes in intracellular Ca^2+^-responses, allowing annexins to impact the host-microbe relationship at many levels. Here, we highlight a few select examples of annexins in the host’s response to pathogens that share a common functional theme. For a more comprehensive overview, we refer to a focused review of this topic^109^.

Several pathogens, including viruses such as human immunodeficiency virus (HIV)^110^, human papillomavirus (HPV)^111^ and bacteria such as uropathogenic *Escherichia coli*^112^ and *Rickettsia*^113^ use AnxA2 on the host cell surface as a receptor to facilitate adhesion and/or entry. For HPV, the initial binding to AnxA2 in complex with its S100A10 ligand is not required for attachment but directs the virus to an endocytic entry pathway that protects it from lysosomal degradation^111^. As a mediator of actin dynamics, AnxA2 in complex with S100A10 is also involved in the striking actin rearrangements observed during infection with enteropathogenic *Escherichia coli* (EPEC)^114^ and *Salmonella*^115^. Thus, AnxA2 alone or in conjunction with S100A10 might direct a pathogen to the appropriate host cell and establish membrane sites that favor pathogen entry. Annexins can also couple the production and assembly of pathogenic virions to specific intracellular organelles, as has been shown for AnxA2 in the replication of hepatitis C virus (HCV) at an HCV-induced membranous web^116^ and for AnxA3 in the assembly of virions at lipid droplets^117^.

Pathogen-elicited immune responses can also be modulated by annexins. For instance, *Yersinia pestis*, the bacterium that causes bubonic plague, detects and destroys immune cells via binding of the needle cap protein of the *Y. pestis* type III secretory system to FPR1. Here, the bacterial protein mimics the FPR1 binding domain of AnxA1^118^. *Helicobacter pylori*, on the other hand, has been shown to bind AnxA5 via the lipid A component of the bacterial outer membrane lipopolysaccharides (LPS) and thereby inhibits LPS-mediated Toll-like receptor 4 (TLR4) activation^119^. Such pathogen-annexin interactions represent a promising starting point for novel annexin-based targeted approaches for drugs that specifically disrupt or enhance the pathogen-annexin interactions in a controlled manner, minimizing non-specific side effects of the drugs. This has been explored experimentally using small molecules to disrupt the AnxA2-S100A10 heterocomplex that efficiently inhibit HPV infection^120^. Moreover, an annexin-mimetic peptide, specifically the AnxA1 N-terminal sequence representing the FPR-binding site, has been exploited to downregulate disease symptoms after dengue virus infection^121^.

Annexin involvement in microbe-host interactions also includes an Anx-mediated reshaping of the organelle or cellular membrane the pathogen relies upon for infection. This is exemplified in the AnxA1-governed regulation of endocytic transport (see above) that is favorable for endosomal trafficking of influenza A virus^122^ and the AnxA6-controlled endolysosomal cholesterol content (see above), which impairs the fusion of enveloped viruses at this entry site^123^. Exploring the role of annexins at such levels of increasing complexity might also uncover new targets for the development of entirely new types of drugs and treatments. This avenue of annexin research could be particularly fruitful based on the wealth of structural and biochemical knowledge on annexins and their ligand interactions.

## Bridging the gap—annexinopathies and annexin-based diagnostic and therapeutic approaches

Altered or mutant expression of several annexins has also been linked to human pathologies and the term ‘annexinopathies‘ has been coined, initially describing the overexpression of AnxA2 in patients suffering from acute promyelocytic leukemia (APML) and the reduction of AnxA5 on placental trophoblasts in antiphospholipid syndrome and preeclampsia patients^124^. AnxA2 is elevated in patients suffering from a hemorrhagic form of APML, associated with the t(15:17) chromosomal translocation. These patients show excessive, leukemia cell-mediated fibrinolysis that could be inhibited by anti-AnxA2 antibodies, in line with a function of AnxA2-S100A10 as cell surface receptor for tPA and plasminogen, triggering plasmin generation and thus fibrin degradation^97,125^. Reduction or displacement of AnxA5, on the other hand, has opposite consequences for trophoblasts where AnxA5 associates with the PS-rich extracellular leaflet of their cell membrane (a special feature of this membrane as extracellular leaflets are typically devoid of PS). This AnxA5 decoration is thought to represent a protective shield inhibiting the initiation of coagulation reactions that would normally occur on exposed negatively charged phospholipids. Antiphospholipid antibodies displace the AnxA5 protective shield and this was linked to pro-thrombotic events in antiphospholipid syndrome patients leading to pregnancy complications^126^. Similarly, reduced AnxA5 gene expression has thromboregulatory consequences, as a haplotype in the AnxA5 gene promoter that decreases its transcription is associated with recurrent pregnancy loss, most likely due to thrombotic placental complications^127^. As a consequence, AnxA5 has gained attention as a therapeutic reagent, exploiting its property to form anti-thrombotic shields on certain activated cell surfaces, which could be relevant in the prevention of atherothrombosis and plaques inflammation in atherosclerotic lesions^128,129^.

The arguably best-known example of PS exposure are apoptotic cells and annexins have been introduced as a diagnostic and highly specific marker of cell apoptosis. This involved mainly AnxA5 and derivatives thereof. Following a thorough characterization of the lipid-binding properties of the protein, labeled AnxA5 species were designed that bind PS-exposing cells and thus detect apoptotic cells not only in cell culture but also in living organisms (for review, see ref. ^130^). As a diagnostic tool for detecting cell apoptosis, AnxA5 has meanwhile been used in thousands of publications and importantly, it has been employed successfully to detect cell apoptosis in patients, e.g., for visualizing the apoptotic tumor response in cancer patients undergoing chemotherapy (for review, see ref. ^131^).

The compelling evidence for the role of AnxA1 in the resolution of inflammation (see above; for review see ref. ^132^) has triggered the use of the AnxA1 protein and its N-terminal peptides (the FPR-binding epitope^89^) in the treatment of inflammatory and infectious disorders in animal models. AnxA1 administration inhibits neutrophil accumulation at sites of inflammation^133^, dampens inflammation in pneumococcal pneumonia^134^, influenza A virus infection^135^, sickle cell disease^136^, and ischemia-reperfusion injury^137^. AnxA1 can also act on human platelets, suppressing classic thrombin-induced inside-out signaling events to decrease integrin αIIbβ3 activation (essential for platelet aggregation) and modify PS to promote platelet phagocytosis by neutrophils, thereby contributing to inflammation resolution^99^. Moreover, AnxA1 N-terminal peptides and small molecule mimetics are being developed as therapeutic tools^94^. Extracellular AnxA1 can also bind to another immune modulator, the tolerogenic dectin-1 receptor on T cells and this constitutes a possibility to generate tolerogenic dendritic cells to counteract autoinflammatory disorders. However, in this case, the interaction is mediated by the core domain, and this property is shared with AnxA5 and AnxA13^138^. The link of AnxA1 to other diseases is also constantly growing. Recent examples are the findings that AnxA1 by targeting the PI3K/AKT/mTOR signaling pathway acts to induce chemoresistance in gastric cancer cells^139^ and that it suppresses the activation of group 2 innate lymphoid cells (ILCs) via regulating the expression of metallothionein genes which modulate zinc homeostasis^140^. As ILCs produce type 2 cytokines and their overproduction can induce allergies, AnxA1 might in the future be used to counteract this.

Whereas the cases discussed above use extracellular annexins as diagnostic and (potential) therapeutic tools, intracellular annexin activities have also been linked to human diseases. A well-known example is AnxA11, which is characterized by an extended N-terminal domain capable of undergoing liquid–liquid phase separation. A genome-wide association study identified a nonsynonymous SNP (coding for a R230C mutation) in the AnxA11 gene to be strongly associated with sarcoidosis, a chronic inflammatory disorder with prime manifestations in the lung^141^. Other mutations, both in the unique N-terminal domain (D40G) and the C-terminal annexin core (R235Q), were identified in patients suffering from amyotrophic lateral sclerosis (ALS) and mutation carriers showed AnxA11 aggregates in spinal cord motor neurons and hippocampal neuronal axons indicative of misfolded and non-functional protein^142^. A recent study showed that AnxA11 forms tethers between lysosomes and RNA granules, thereby mediating intracellular transport of the RNA granules on microtubule-associated lysosomes (Fig. 2). The above-mentioned ALS mutations in AnxA11 disrupt the interaction with lysosomes and thereby inhibit RNA granule transport suggesting that impaired neuronal transport of RNA to sites of local protein synthesis could be a factor in the development of ALS^143^. As alluded to earlier, RNA binding might be a more general feature of annexin biology and could open up another interesting field of annexin research^19^.

Although some annexinopathies have been known for many years, the link between annexins and human diseases (Table 1) is only beginning to emerge yet might be valuable in progressing toward a personal medicine approach. Are other annexin gene sequence variations associated with human pathologies? Will pathological single nucleotide polymorphisms (SNPs) affect common or unique annexin functions, resulting in annexinopathies?

## Perspectives—toward a holistic understanding of annexins

### A unifying picture of annexin function

**Fig. 4:**
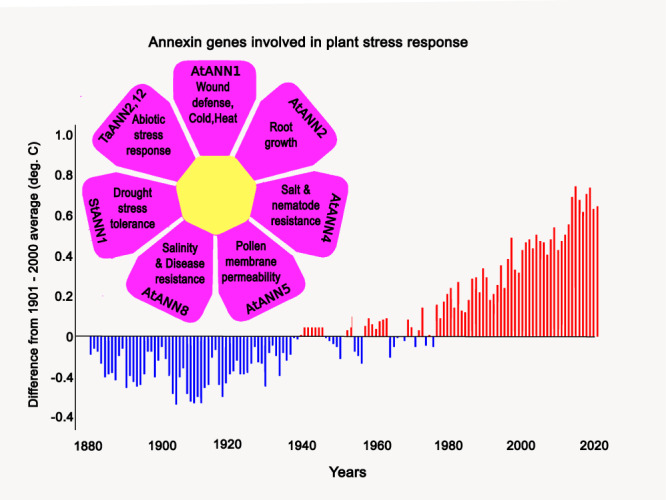
Plant annexins, stress response and global warming. Graphic showing a selection of the putative functions of plant annexins highlighted in the different petals. The relevant annexin gene names shown are: *AtANN1x* (*Arabidopsis thaliana*—Thale cress), *STANN1* (*Solanum tuberosum*—potato) and *TaANN2 & 12* (*Triticum aestivum*—common wheat). As plant annexins play an important role in adaptation to drought and salt stress, conditions that will be increasingly met in future climates, the petals are superimposed on a bar chart indicating global land temperature change (plotted as a difference from average between 1880 and 2020; source: NOAA climate.gov; graph based on data from the National Centers for Environmental Information).

The common denominator of annexin functions is their Ca^2+^-regulated membrane binding which is exploited by cells when responding to stress that raises cellular Ca^2+^. This is most evident upon plasma membrane injury, where annexins sense the supraphysiological elevated Ca^2+^ levels as danger signals. They link this to a cellular, membrane-centered response involving membrane sculpting, aggregation, and fusion of membrane to seal the wound. While this process involves several annexins with varying Ca^2+^ sensitivities, subtle Ca^2+^ alterations are sensed by different annexins with graded Ca^2+^ sensitivities. This could explain why while many annexins respond to cell membrane injury, only the most Ca^2+^-sensitive annexin, AnxA2, is involved in hormone-evoked exocytotic responses triggered by physiological Ca^2+^ elevations. Similar to animal annexins, plant annexins also respond to environmental signals that raise intracellular Ca^2+^ by altering membrane trafficking and cytoskeletal dynamics^8^. Plant annexins participate in abiotic stress responses such as the Ca^2+^ dependent regulation of salt, drought, and heat tolerance in Arabidopsis, stressors that will increase due to global warming (Fig. 4 with functions of different plant annexins depicted in the different petals)^9,144–146^. Thus, response to stress scenarios encoded by elevation of intracellular Ca^2+^ is a general annexin feature that is evolutionarily conserved and mediated by the Anx core. Other elements of the annexin molecules, mostly found in the unique N-terminal domains, link this general property to specific functions, such as the formation of complexes with cargo-binding S100 proteins for plasma membrane targeting (AnxA2), with cell polarity proteins to instruct spindle orientation and with RNA for RNA granule transport (AnxA11), to name a few.

### The major unanswered questions

**Fig. 5:**
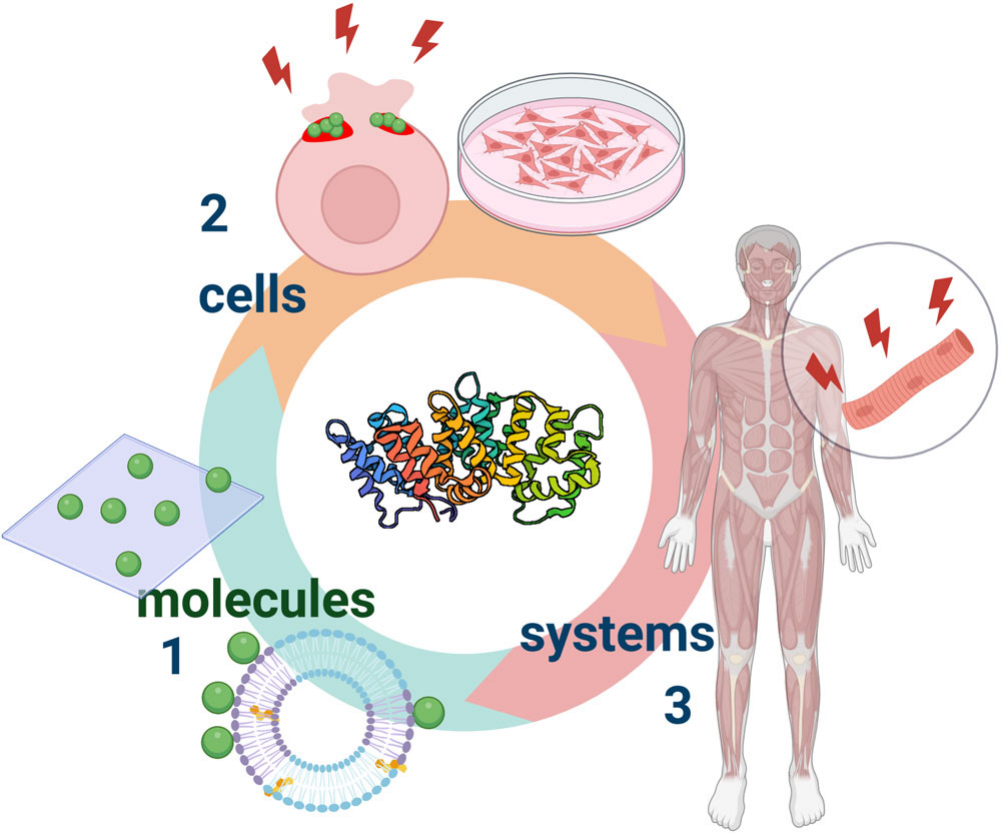
From molecules to cells to systems—annexin research across scales. Nanotechnological tools such as solid-supported or vesicular lipid bilayers are employed to assess molecular principles governing the interaction of annexins with membranes (1). Cell culture models in 2D and 3D are exploited to analyze annexin functions at the cellular level (2). These approaches together with work in whole organisms will result in a systemic understanding of annexin function leading to the exploitation of annexin properties in the diagnostics and possibly the treatment of human pathologies (3) (see the text for details). Green spheres represent annexins and red lightning flashes should highlight stress that impacts membrane integrity. Generated with BioRender.com.

Ongoing effort remains focused on understanding annexin functions across scales—from molecules to cells to systems (Fig. 5). What is missing to date is a more systematic approach to annexin biology. At the molecular level, comparison of their specific lipid-binding preferences can be assessed by solid-supported or vesicular lipid bilayers, in combination with biochemical approaches to study annexin-containing supramolecular protein–protein and protein–membrane complexes, and by employing model membranes to examine the function of regulated membrane binding as the most central one in annexin biology. We envision significant progress resulting from such studies to generate novel useful compounds, such as small molecules, to interfere with or mimic annexin domains to manipulate cellular functions. Fragment-based approaches might also be applicable to decompose the annexins into smaller structural units (mini-annexins) to identify the minimum annexin motifs that retain the biological activity. This can be aided by computational approaches, which could lead to annexin-derived peptides with altered and fine-tuned properties.

Concepts and insights obtained at the molecular level can then be put to the test in cells. While most of our annexin knowledge comes from classical cell biological studies at this level, future approaches have to further characterize these and yet unexplored roles of annexins in their native environment. This can be achieved by, e.g., proteomic and functional studies, 2D and 3D cell systems, including organoids generated from healthy and diseased (patient-derived) tissues. Activity screens of annexin expression controlled by their own native promoters will reveal their regulation pattern and parameters of induction. Together with the specific localizations of the different annexins such studies are needed to determine their specific sites of action to make clear whether different members have specialized or generalized roles or if in fact they are functionally redundant. These approaches will benefit from the availability of CRISPR-based genome editing for loss-of-function approaches and targeted mutations, and might be efficiently combined with high-throughput phenotypic screens.

The combination of approaches rooted in cell biology, pharmacology, biochemistry, and chemical biology offers a great future for annexin research to truly define annexin function at the systems level and unlock the potential of these fascinating molecules.
